# Microbial Dynamics of Yogurts with Different Starter Cultures Under an In Vitro Gastrointestinal System Using 16S rRNA Sequencing

**DOI:** 10.3390/foods15112043

**Published:** 2026-06-05

**Authors:** Merve İnce-Palamutoğlu, Recep Palamutoğlu, Murat Baş

**Affiliations:** 1 Department of Nutrition and Dietetics, Faculty of Health Sciences, Afyonkarahisar Health Science University, Afyonkarahisar 03030, Türkiye; merve.palamutoglu@afsu.edu.tr (M.İ.-P.); recep.palamutoglu@afsu.edu.tr (R.P.); 2 Department of Nutrition and Dietetics, Graduate School of Health Sciences, Acibadem Mehmet Ali Aydınlar University, Istanbul 34752, Türkiye; 3 Department of Nutrition and Dietetics, Faculty of Health Sciences, Acibadem Mehmet Ali Aydınlar University, Istanbul 34752, Türkiye

**Keywords:** probiotic, yogurt, in vitro gastrointestinal system, 16S rRNA sequencing

## Abstract

Background: The aim of this study was to compare microbial diversity and compositional changes under digestive stress in yogurts produced using different culture strategies in a dynamic in vitro gastrointestinal system. Methods: Yogurts produced with probiotic starter cultures, standard yogurt cultures, and probiotic sachet supplementation were evaluated using a dynamic in vitro gastrointestinal system including mouth, stomach, and small intestinal phases under controlled pH, temperature, and digestion conditions. Microbial diversity and compositional changes before and after the in vitro gastrointestinal simulation were determined using a 16S rRNA amplicon-based Oxford Nanopore sequencing approach, and the resulting data were analyzed using bioinformatics, alpha-diversity, and beta-diversity metrics. Results: Probiotic sachet addition significantly increased microbial species richness, alpha diversity, and community balance compared to starter culture yogurts (Shannon and Inverse Simpson, *p* < 0.05). In vitro gastrointestinal system led to a reduction to a Firmicutes-dominant core microbiota in all samples; in contrast, beta diversity and PCoA analyses showed that the post-digestive microbial structure of sachet-supplemented yogurts was significantly different (PERMANOVA, *p* = 0.001). Conclusions: Consequently, it was demonstrated that adding probiotic sachets to yogurt increases the diversity and resilience of beneficial bacteria throughout the digestive tract, making the product a more robust and functional food for health.

## 1. Introduction

Yogurt is a fermented dairy product, and the strains *Lactobacillus delbrueckii* subsp. *bulgaricus* and *Streptococcus thermophilus* are generally used in its production [1]. These two bacteria are the traditional starter cultures responsible for the fermentation process of yogurt. These bacteria can be live microorganisms with probiotic properties, but this depends on criteria such as the yogurt’s production process, storage conditions, viability until the expiration date, and scientific demonstration of specific health effects [2].

The Food and Agriculture Organization (FAO) and the World Health Organization (WHO) have recently defined probiotic bacteria as “live microorganisms that, when consumed in sufficient numbers, confer beneficial health effects on the host’s gastrointestinal system” [3]. To obtain these benefits, probiotic microorganisms must be consumed regularly in sufficient numbers and be present in the digestive system along with stomach acid, bile salts, and various enzymes. Probiotic bacteria must be able to maintain their viability under these conditions, reach and colonize the intestines in sufficient quantities, and maintain their viability throughout the product’s shelf life [4]. When they reach the intestines, probiotic bacteria must have a minimum of 10^6^–10^8^ colony-forming units (cfu) per g or mL [5]. The main groups of probiotic microorganisms consist of lactic acid bacteria, primarily including *Bifidobacterium* and *Lactobacillus* species, as well as the yeast *Saccharomyces boulardii* [6]. The literature reports that some yogurts enriched with probiotic strains—particularly *Lactobacillus acidophilus* and *Bifidobacterium* species—can exhibit probiotic effects, as they more clearly meet this definition [7,8].

The growing interest in modifying the matrix and structural properties of foods to optimize digestion and absorption behaviors for health benefits necessitates the implementation of numerous studies on food digestion in the digestive system [9]. In vivo studies on humans are considered the ideal approach for obtaining reliable and accurate data under gastrointestinal conditions. However, the technical, financial, and ethical feasibility of human clinical studies is questionable, and reproducibility is low due to individual differences [10]. The complex processes involved in gastrointestinal studies on humans and/or animals make these studies technically challenging and costly, and ethical constraints further complicate the conduct of in vivo studies [11]. These limitations have prompted researchers to develop reliable in vitro models that closely simulate the human gastrointestinal system, incorporating digestive enzymes as well as the mouth, stomach, and intestinal phases, while accounting for factors such as digestion time and pH. In vitro models offer a valuable alternative to human and animal studies, providing greater flexibility, accuracy, and reproducibility [12]. In vitro gastrointestinal models are designed to simulate the human digestive system using cone-, funnel-, or cylinder-shaped devices, where mechanical forces on food are generated through rhythmic peristaltic contractions [10]. Depending on the study’s purpose, these models may include the phases of the mouth, stomach, and small intestine. Each phase is treated with simulated digestive fluids at body temperature for a defined period, while the pH is maintained at a constant level using appropriate buffer solutions [13].

16S rRNA amplicon sequencing enables the identification of microbial taxa, community diversity, and population structure through direct DNA extraction from samples without the need for culturing microorganisms [14]. Microbiota analysis can generally be performed using two main sequencing approaches. The first approach aims to reveal the complex relationships that exist among members of the studied microbiota. The second approach seeks to create a database comprising thousands of different genes obtained from various studies [15]. Microbial diversity can be evaluated using both targeted amplicon sequencing and shotgun metagenomic sequencing approaches. Before selecting a sequencing strategy, the characteristics of the microbial community, the study objectives, budget limitations, and the required technical expertise should be carefully considered. For example, targeted amplicon sequencing is suitable for identifying lactic acid bacteria in fermented food microbiota, whereas shotgun metagenomic sequencing enables comprehensive characterization of the entire microbial community within a sample [14].

In recent years, community-level microbial profiling approaches—particularly 16S rRNA amplicon sequencing—have enabled high-resolution characterization of microbial communities in fermented foods. Similarly, in vitro gastrointestinal system simulation models offer controlled and reproducible platforms for studying digestion-associated stresses, including acidity, bile salts, and enzymatic activity. Despite these technological advances, there is limited evidence integrating dynamic gastrointestinal system models with 16S rRNA-based microbial profiling to evaluate microbial survival in yogurts produced using different probiotic culture strategies.

Building on this gap, the present study aimed to investigate microbial dynamics and compositional shifts in yogurts produced with probiotic starter cultures, standard yogurt culture, and probiotic sachet supplementation under dynamic in vitro gastrointestinal conditions. Using 16S rRNA amplicon sequencing, we investigated the impact of culture type and digestive stress on microbial diversity, species distribution, and dominant taxa at multiple taxonomic levels. It was hypothesized that probiotic sachet supplementation would enhance microbial diversity and improve microbial persistence during simulated digestion compared with conventional starter cultures.

## 2. Materials and Methods

### 2.1. Materials

This study used milk samples obtained from the same lot and expiration date for yogurt production. For the first six groups (A, B, C, D, E, and F), commercially available probiotic starter culture sachets were added individually to the milk according to the manufacturer’s instructions on the package. Each starter culture sachet contained at least 1 × 10^9^ cfu of viable microorganisms, as stated in the product declaration. Starter cultures A, B, and C contained *Streptococcus thermophilus*, *Lactobacillus acidophilus*, *Lactobacillus bulgaricus*, and *Bifidobacterium animalis* subsp. *lactis*. Starter cultures D and F consisted of *Streptococcus thermophilus*, *Lactobacillus acidophilus*, *Lactobacillus bulgaricus*, *Lactobacillus casei*, *Bifidobacterium infantis*, *Bifidobacterium longum*, and *Bifidobacterium bifidum*. Starter culture E contained *Streptococcus thermophilus*, *Lactobacillus acidophilus*, *Lactobacillus bulgaricus*, *Bifidobacterium animalis* subsp. *lactis*, *Lactobacillus casei*, *Lactobacillus rhamnosus*, *Lactobacillus paracasei*, and *Bifidobacterium infantis*.

For the following six groups (G, H, J, K, L, and M), yogurt was produced using 20 g of the same standard starter culture in all samples, and five different probiotic sachets were individually added to the standard starter culture during production. According to the product declaration, each probiotic sachet contained at least 5 × 10^9^ cfu of viable microorganisms. Probiotic sachet H contained *Lactobacillus rhamnosus* GG. Probiotic sachet J contained *Bifidobacterium animalis* subsp. *lactis* and *Lactobacillus acidophilus*. Probiotic sachet K contained only *Bifidobacterium animalis* subsp. *lactis*. Probiotic sachet L consisted of *Lactobacillus acidophilus*, *Lactobacillus rhamnosus*, *Lactobacillus casei*, and *Bifidobacterium bifidum*. Probiotic sachet M contained *Enterococcus faecium*, *Lactobacillus acidophilus*, *Lactobacillus rhamnosus*, *Bifidobacterium longum*, and *Bifidobacterium bifidum*.

All samples were incubated at 42 °C for 8 h (Daihan-ThermoStable IG-105, DAIHAN Scientific Co., Ltd., Wonju, South Korea). Following fermentation, the samples were allowed to reach room temperature and stored refrigerated until in vitro gastrointestinal digestion analysis was performed. No company names were disclosed in this study, and no statements implying or identifying any commercial company were included. All products were coded alphabetically, and the results were presented in alphabetical order.

### 2.2. Methods

#### 2.2.1. Establishment of Dynamic In Vitro Gastrointestinal Model

This dynamic in vitro digestive model simulates the mouth, stomach, and small intestine sections. A temperature-controlled water bath was used for the mouth section, and a double-jacketed reaction vessel kept at 37 °C was used for the stomach and small intestine sections. Temperature and pH were continuously monitored. Digestion time was determined as 5 min in the mouth section, 2 h in the stomach section, and 2 h in the small intestine section. Secretion flow rates were controlled with adjustable peristaltic pumps. The pH balance of the stomach and small intestine sections was provided with 1 M Sodium Hydroxide (NaOH) and 1 M Hydrochloric Acid (HCl).

Mucin, α-amylase, and a 40% NaOH solution were used to simulate salivary secretion. The saliva secretion was prepared at pH 6.9 by mixing 2 g/L α-amylase, 1 g/L mucin, 25 mL of 0.3 M CaCl_2_, and 975 mL of distilled water at 20 °C. Simulated saliva (0.05 mL/g sample) was added to the mouth environment at a flow rate of 5 mL/min, and all reagents were incubated at 37 °C for 5 min. Gastric fluid simulation involved mucin and pepsin enzymes to promote acid denaturation of digested foods, with HCl used to activate pepsin. The gastric buffer solution consisted of 2.2 g/L KCl, 6.2 g/L NaCl, 1.2 g/L NaHCO_3_, and 0.22 g/L CaCl_2_. To simulate gastric secretion, 3700 ppm/L pepsin and 23 g/L mucin were dissolved in the sterile gastric buffer solution. Simulated gastric secretion (0.05 mL/g sample) was added to the stomach reactor at a flow rate of 0.25 mL/min. After digestion in the mouth phase at pH 6.9, samples entered the stomach reactor at a flow rate of 100 mL/min. Gradual acidification to pH 2.5 was achieved by adding 0.2 mL of 1 M HCl and 0.695 mL of water, with the HCl flow adjusted to 3.5 mL/min until reaching pH 2.5 and then reduced to 0.9 mL/min to simulate gastrin inhibition. Gastric digestion was maintained for 2 h at 37 °C. A double-jacketed reactor integrated with a circulating water bath ensured constant temperature throughout digestion. Pancreatin and bile salts were used to simulate small intestinal fluid. The small intestine buffer solution was prepared with 0.6 g/L KCl, 5.0 g/L NaCl, 0.25 g/L CaCl_2_, 1 g/L pancreatin, 12 g/L bile salts, and 1 M NaHCO_3_ dissolved in the small intestine buffer solution to simulate intestinal secretion. Simulated intestinal secretion (0.25 mL/g sample) was introduced at a flow rate of 3 mL/min. Samples were transferred from the stomach reactor (pH 2.5) to the small intestine reactor at 100 mL/min over 20 min. The pH was gradually increased to 6.9 by adding 1 M NaOH at a flow rate of 0.65 mL/min. Intestinal digestion was maintained for 2 h at 37 °C. Temperature, digestion times, secretion compositions, and flow rates were set based on established gastrointestinal simulation protocols from the literature [11,13].

Alfa-amylase, mucin, bile salt, pancreatin, and pepsin used in the study were obtained from Sigma-Aldrich (Saint Louis, MO, USA), CaCl2, NaCl, NaHCO3, and NaOH were obtained from AFG Bioscience (Northbrook, IL, USA), KCl was obtained from TEKKİM (Bursa, Türkiye), and HCl was obtained from Honeywell (Raunheim, Germany).

#### 2.2.2. DNA Extraction

DNA extractions of the samples were performed using a physical disruption method optimized for bacteria (ZymoBIOMICS DNA Miniprep Kit, E4300, Zymo Research Co., Irvine, CA, USA). DNA quantity and quality were assessed using a Nanodrop OneC spectrophotometer, and the measurements were ensured to fall within the reference ranges provided by Oxford Nanopore Technologies (Oxford, UK) (260/230: 1.8–2.0; 260/280: 1.6–1.8). Samples outside these ranges were subjected to re-extraction.

#### 2.2.3. Sequencing

The sequencing library was prepared according to the ONT 16S Barcoding Kit (SQK-16S114-24) manual (6S_9199_v114_revG_01Sep2025). Following library preparation, 24 samples and 12 chassis DNA isolates were loaded onto a FLO-MIN114 (r10.4.1) flow cell, and sequencing was conducted on the MinION Mk1B device. Oxford Nanopore Technology (ONT) was selected because its long-read sequencing capability enables full-length 16S rRNA gene analysis, providing improved taxonomic resolution compared with short-read sequencing approaches. Full-length sequencing facilitates more accurate discrimination among closely related bacterial taxa and allows species-level characterization of complex microbial communities in fermented food matrices. In addition, ONT provides flexibility for rapid library preparation and comprehensive profiling of diverse microbial populations.

#### 2.2.4. Bioinformatic Analysis

Raw signal files (*.pod5) obtained from sequencing using the MinKNOW GUI software (v. 25.09.16) were basecalled with Dorado (v. 1.0.1) using the “hac” model to generate *.fastq.gz files. Raw and post-validation sequence quality assessments were performed with FastQC (v. 0.12.1). Adapter, primer, and quality trimming were carried out using bbduk (v. 39.06), and consensus sequence generation and error correction were completed with medaka (v. 2.1.1). Filtering steps were performed using magicblast (v. 1.7.0) and samtools (v. 1.16.1), while final taxonomic annotation was conducted using blastn (v. 2.15.0). The databases used in this pipeline included NCBI RefSeq, a curated databank provided by Epigenetiks A.Ş., and the NCBI Entrez database (as of 12 October 2025).

### 2.3. Statistical Analysis

Relative abundance tables were generated from the raw read count tables using the TSS (total sum scaling) normalization method, and subsequent analyses were performed on these tables. Descriptive (data distribution, mean, median, minimum, maximum, and variation) and exploratory analyses (hypothesis testing, diversity analyses) as well as visualizations were carried out using the R statistical programming language (v. 4.5.2).

Samples were analyzed by dividing them into two different two-group comparisons. The first comparison involved yogurts produced using probiotic yogurt starter sachets versus yogurts produced with standard yogurt culture but supplemented with probiotic sachets. The second comparison included analyses of the in vitro gastrointestinal system at baseline (mouth phase, 0 min) and final output (small intestine, 120 min).

In the first analysis set, since samples were not paired-longitudinal, different hypothesis tests were applied. For normally distributed data, *t*-tests and ANOVA were used, whereas for non-parametric distributions, Mann–Whitney U (Wilcoxon Rank Sum) and Kruskal–Wallis tests were applied. The FDR (false discovery rate) Benjamini–Hochberg algorithm was used as the post hoc *p*-value correction method. In the second analysis set, the pre- and post-simulation states of the samples were compared. The distribution of the data was assessed using the Shapiro–Wilk and Kolmogorov–Smirnov tests, and the appropriate hypothesis tests were selected based on these results. For normally (Gaussian) distributed paired groups, paired *t*-tests and ANOVA (for more than two groups) were applied; for non-normal distributions, Wilcoxon (Wilcoxon Signed Rank) and Friedman tests (for more than two groups) were used. Visualizations were generated using the R packages ggplot2, rColorBrewer, and openxlsx, and the significance threshold was set at 0.05.

For diversity analyses, two major approaches were used: alpha and beta diversity (R packages: phyloseq, vegan, microbiome, ggplot2, and rColorBrewer). Alpha diversity considers the diversity within each individual sample. After rarefaction, several indices were computed: observed diversity, Shannon, and Simpson (Inverse Simpson) indices. Observed diversity reflects the number of taxa after rarefaction; the Shannon index incorporates both richness and evenness; and the Simpson index places greater emphasis on dominant taxa, allowing identification of whether differences in diversity stem from changes in abundance distribution. Comparisons were performed using the relevant hypothesis tests described above. Depending on the number of test repetitions, *p*-value correction was applied.

Beta diversity reflects differences between sample groups and is calculated based on dissimilarity distances. Two beta diversity indices were assessed: the Jaccard index and Bray–Curtis dissimilarity. Jaccard matrices were constructed using presence–absence data, whereas Bray–Curtis incorporated abundance information. The resulting matrices were visualized using two approaches: heatmaps and PCoA plots. Heatmaps were generated together with UPGMA clustering to illustrate sample relatedness via a cladogram. Principal Coordinate Analysis (PCoA), a dimension reduction method, constructs each axis using combinations of taxa, and each axis is accompanied by its explained variance. Statistical comparisons of beta diversity groups were performed using the non-parametric PERMANOVA (permutational ANOVA) test with 999 permutations. It should be noted that PERMANOVA may yield significant *p*-values even for overlapping groups in cases of heterogeneous distribution, and therefore 95% confidence ellipses of distributions should be examined carefully during interpretation.

## 3. Results

Yogurt samples produced using probiotic starter cultures from different commercial brands were coded as A, B, C, D, E, and F, while the sample produced with a standard yogurt culture was coded as G. Yogurts produced by adding a probiotic sachet to the standard yogurt culture during production were coded as H, J, K, L, and M. In the dynamic in vitro gastrointestinal system, samples collected at the beginning of the simulation (mouth phase, 0 min) were coded as “0”, and those collected at the end of the simulation (small intestine, 120 min) were coded as “2”.

**Figure 1:**
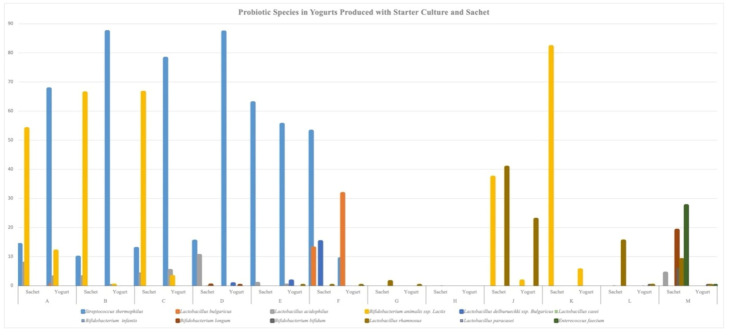
Comparison of probiotic species in yogurts produced using probiotic starter culture and probiotic sachets. Light blue bars represent *Streptococcus thermophilus*, dark gray bars represent *Bifidobacterium infantis*, orange bars represent *Lactobacillus bulgaricus*, burgundy bars represent *Bifidobacterium longum*, gray bars represent *Lactobacillus acidophilus*, black bars represent *Bifidobacterium bifidum*, yellow bars represent *Bifidobacterium animalis* subsp. *lactis*, brown bars represent *Lactobacillus rhamnosus*, dark blue bars represent *Lactobacillus delbrueckii* ssp. *bulgaricus*, dark brown bars represent *Lactobacillus paracasei*, light green bars represent *Lactobacillus casei*, and green bars represent *Enterococcus faecium*.

According to Figure 1, the dominant species in yogurts produced with probiotic starter cultures (A–F) were *Streptococcus thermophilus* and *Lactobacillus delbrueckii* subsp. *bulgaricus*, which represent the characteristic microbiota of yogurt fermentation. These two species constituted the majority of the microbial composition in most samples. In addition, *Bifidobacterium infantis* and *Bifidobacterium longum* were detected at relatively low abundance in selected samples (A, B, and E). In contrast, yogurts supplemented with probiotic sachets (H–M) exhibited a more heterogeneous microbial composition. Species including *Lactobacillus casei*, *Lactobacillus paracasei*, *Lactobacillus rhamnosus*, *Bifidobacterium animalis* subsp. *lactis*, and *Enterococcus faecium* were detected at varying abundances among samples. Higher relative abundance of *Bifidobacterium animalis* subsp. *lactis* was observed in samples K and L, whereas *Enterococcus faecium* was more abundant in sample M. Overall, probiotic sachet supplementation resulted in broader microbial composition and increased heterogeneity among yogurt samples.

Alpha diversity analyses at the genus level (Observed richness, Chao1, ACE, Shannon, and Inverse Simpson indices) demonstrated differences among CN, G0, and G2 samples (Table 1). In starter culture yogurts (CN), the number of genera ranged from 53 to 224, with the highest value observed in sample CNA (224). G0 samples exhibited moderate richness (49–126 genera), whereas G2 samples displayed a more heterogeneous distribution, with increased richness in some samples, particularly G2H and G2M. Significant differences were observed only for ACE (*p* = 0.01) and ACE standard error (*p* = 0.03), indicating variation in the distribution of rare taxa among groups. Shannon values ranged from 1.21 to 5.60, suggesting moderate-to-high diversity across samples. Samples CNM (5.41), G0J (5.495), and G2G (5.607) exhibited relatively higher Shannon values, whereas lower Inverse Simpson values in several samples indicated a greater dominance of specific taxa. Overall, the findings suggest that gastrointestinal simulation altered microbial richness and community structure in a sample-dependent manner.

Observed values for the PS group ranged from 47 to 129, while for the SA group, this range was determined as 80 to 240 (Table 2). The highest enrichment value was obtained in the SA02 sample (Observed = 240; Chao1 = 366). This indicates that adding different types of probiotics to the yogurt matrix in the sachet significantly increased microbial species diversity. The ACE index differed significantly (*p* = 0.05), supporting higher species richness in the sachet group. Regarding the Shannon index, a wide range was observed: 3.40–5.44 in the PS group and 1.21–5.59 in the SA group. The moderate-to-high Shannon values observed in the PS group (especially PS06: 5.44) indicate that starter culture yogurts offer a more balanced microbial distribution, despite their low species richness. In other words, although there are distinct dominant species in PS yogurts, the community structure appears homogeneous and stable. In the SA group, high Shannon values (5.495–5.599) in samples such as SA02 and SA07 indicate the potential to create a rich, balanced microbiota in products with added sachets. In contrast, the low Shannon values in some samples, such as SA01 and SA06, suggest that the species in the sachets may have been rendered ineffective or suppressed by competitive dynamics within the yogurt. The distribution of Inverse Simpson values also supports this result; for example, in SA09 (InvSimpson = 6.832), the community is not tightly suppressed by a dominant species, and interspecific distributions are quite balanced. The statistically significant Shannon (*p* = 0.04) and Inverse Simpson (*p* = 0.03) indices indicate a real ecological divergence in microbial community balance between the two groups. In the SA group, high alpha diversity indicates that different species in the sachets were able to adapt to the yogurt matrix despite competitive conditions. However, samples with low Shannon values, such as SA01 and SA06, demonstrate the sensitivity of some probiotic species to environmental conditions and the variability in microbial success rates across products. Samples with high Shannon values, particularly SA07 and SA02, indicate that probiotic products containing different species may provide broader probiotic effects. The extremely high values of the Chao1 and ACE indices, especially in the SA group, indicate that genetic traces of rare species are more frequently detected in samples with added sachets. This suggests that 16S rRNA amplicon sequencing analysis captures different probiotic mixtures in the sachets, species with low abundance in the yogurt matrix, and pre-digestive genetic signals. Starter culture yogurts are microbiologically more homogeneous, with a limited number of species but a stable community balance, offering greater control in industrial production. Traditional yogurts with added sachets, on the other hand, have higher species richness, broader functional capacity, and a richer probiotic diversity profile. The significant differences in Shannon and Inverse Simpson indices strongly suggest that the microbial ecology of PS and SA yogurts are two biologically different ecosystems.

**Figure 2:**
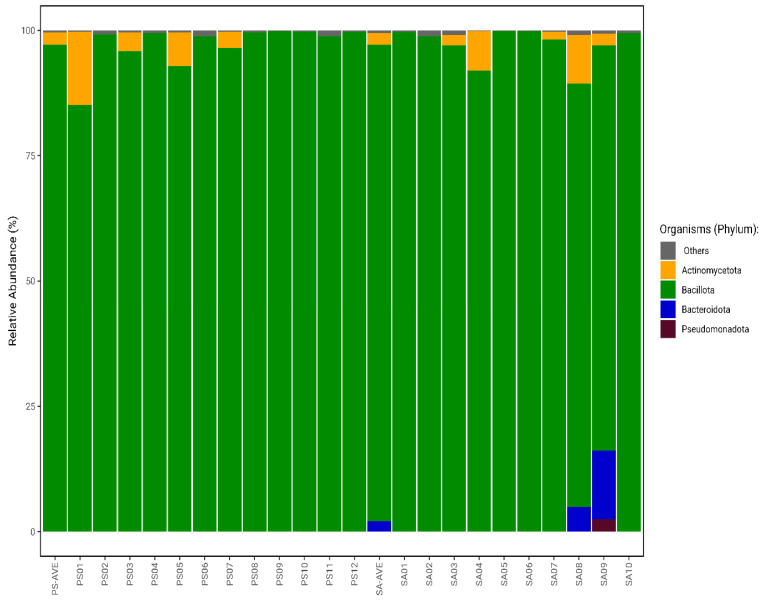
Phylum-level microbial composition of PS and SA yogurt samples.

As shown in Figure 2, *Firmicutes* (*Bacillota*) were dominant across all samples, whereas *Actinomycetota* and *Bacteroidota* were primarily detected in yogurts enriched with probiotic sachets. For both groups, the majority of the microbial composition consisted of *Firmicutes* (*Bacillota*), which is consistent with the typical microbial structure of yogurt, as major fermentative genera such as *Lactobacillus* and *Streptococcus* belong to this phylum. In the PS group, phylum distribution was relatively homogeneous, with *Firmicutes* representing approximately 95–100% of the microbial composition in most samples. Only a few samples, including PS05, PS07, and PS10, showed relatively low proportions of *Actinomycetota* (<10%) and *Bacteroidota*. In contrast, although Firmicutes remained the predominant phylum in the SA group, greater phylum-level diversity was observed in several samples. Low-level increases in *Bacteroidota* were detected in SA07 and SA08, whereas relatively higher proportions of *Actinomycetota* and *Bacteroidota* were observed in SA09 and SA10. In addition, low-abundance taxa belonging to *Pseudomonadota* and the “Others” category were detected in SA11. The presence of *Actinomycetota* in the SA group may be associated with the inclusion of *Bifidobacterium* species in probiotic sachets. Overall, PS samples exhibited a relatively homogeneous microbial composition, whereas SA samples displayed broader phylum-level variation.

**Figure 3:**
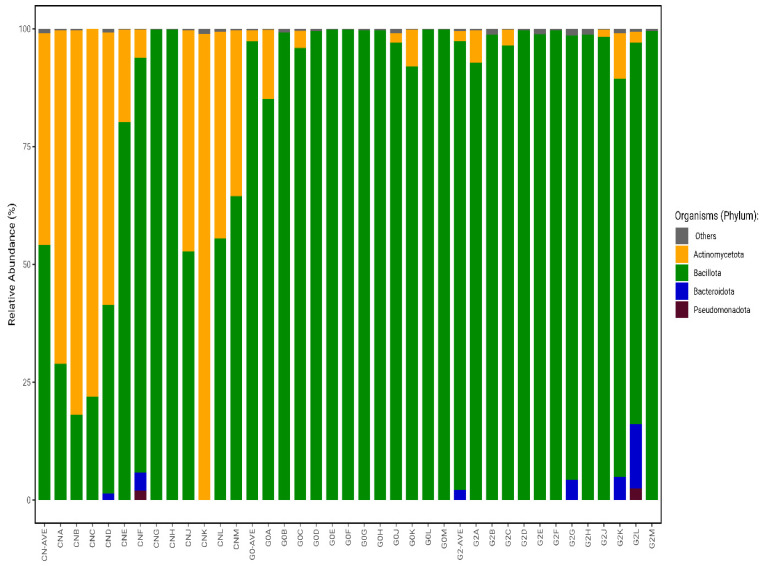
Phylum-level microbial composition in gastrointestinal simulation samples (GO vs. G2).

Figure 3 highlights the major transition in microbial structure from the mouth entry stage (GO) to the intestinal exit stage (G2). This emphasizes the dynamic changes in phylum composition during gastrointestinal transit. G2 samples show a strong phylum-level contraction dominated by *Firmicutes*. This pattern is consistent with selective survival under acidic, enzymatic, and bile salt stress. GO samples (GOA–GOM) display high phylum heterogeneity. *Firmicutes* dominate, but additional phyla, such as *Actinomycetota* (particularly *Bifidobacterium*), *Bacteroidota*, and *Pseudomonadota*, are also detected. This indicates that the initial yogurt microbiota has a wide range of probiotic diversity. High levels of *Actinomycetota*, particularly in GOC, GOD, GOE, GOF, GOG, and GOH, indicate the presence of Bifidobacterium spp. from the probiotic sachet. *Bacteroidota* signals also appear at low levels in GOE, GOK, and GOL. These species are not usually dominant in yogurt fermentation but may be of environmental origin. Small amounts of *Pseudomonadota* were detected in GOH and GOJ; these could be low-abundance environmental DNA traces, equipment contamination, or secondary species from sachet components. The table shows that the yogurt microbiota before simulation is highly rich, heterogeneous, and multi-phylum. A significant reduction in phylum diversity was observed in G2 samples (G2A–G2M). Firmicutes became dominant at levels of 95–100% in almost all samples. *Actinomycetota* was only preserved at low levels in a few samples, such as G2H and G2L. *Bacteroidota* and *Pseudomonadota* signals were largely lost. In some samples (e.g., G2J, G2K, G2M), a single-phylum composition reduced entirely to *Firmicutes* was observed. Most *Actinomycetota* species were eliminated. The low bile salt tolerance and oxygen sensitivity of *Bifidobacterium* species are consistent with this. *Bacteroidota* and *Pseudomonadota* have completely disappeared. These species are generally guest species with low abundance rather than being permanent in yogurt. *Firmicutes* (especially the *Lactobacillus* group) have become overly dominant. These species provide an advantage in the gastrointestinal process thanks to their stress tolerance, acid resistance, and enzymatic adaptation mechanisms. However, the *Firmicutes* dominance observed in post-digestion samples may not solely reflect selective microbial survival. Methodological factors associated with the in vitro gastrointestinal model and sequencing-based microbial profiling, including differences in DNA extraction efficiency, amplification bias, and the inability of the model to fully reproduce physiological gastrointestinal conditions, may also contribute to the observed taxonomic distribution. Therefore, the enrichment of *Firmicutes* likely reflects a combination of biological selection and methodological influences rather than a single mechanism.

The elimination of most *Bifidobacterium* species during the G2 phase may limit the transport of the sachet supplement to the distal intestine. However, since *Lactobacillus/Lacticaseibacillus* species have a high colonization potential in the gastrointestinal system, a beneficial effect can be expected, especially on immune and digestive functions. The change in the phylum-level profile from G0 to G2 suggests that the 16S-ONT pipeline can capture the effects of biological selection. The narrowing of diversity is a result of biological selection, as different levels of phylum conservation are observed across samples. The low levels of *Actinomycetota* in samples such as G2H suggest the presence of partially resistant species. The disappearance of *Bacteroidota*/*Pseudomonadota* signals in G2 samples indicates that the pipeline does not over-sort and is highly specific.

**Figure 4:**
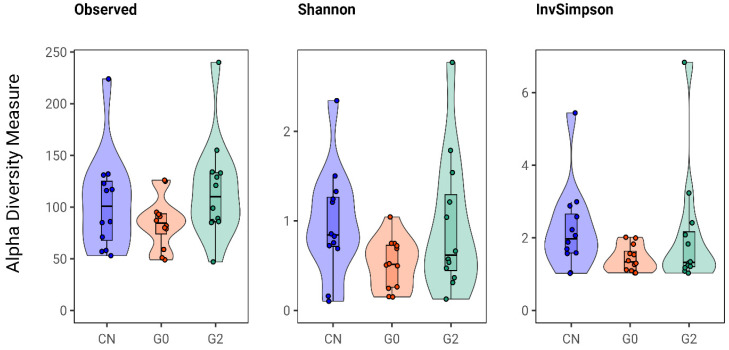
Genus-level alpha diversity violin plot for CN, GO, and G2 samples.

The violin plots in Figure 4 show the distributions of the Observed, Shannon diversity, and Inverse Simpson indices. CN samples show the highest equilibrium and richness, while GO samples reflect early-stage contraction under gastrointestinal stress, and G2 samples show heterogeneously reorganized communities. The results reveal significant variations in microbial reorganization occurring at the beginning product type (CN), gastrointestinal model entry (GO), and end-of-digestion (G2) stages. According to the Observed index, there is a clear differentiation among the three groups, with the CN group exhibiting the widest species richness distribution (approx. 50–220). The GO group has significantly lower species richness than the CN group (approx. 40–100). This indicates that even in samples with added probiotic sachets, a contraction in species number occurs in the G0 stage due to factors such as matrix-induced elimination, pH, oxygen levels, and competition in the yogurt. The G2 group has a larger species count (approximately 40–250) than both the GO and CN groups, but its distribution is much more heterogeneous. In some samples (e.g., G2H), the species count increases dramatically, whereas in others (e.g., G2C, G2E) it is limited. This suggests that in the G2 group, two intrinsic mechanisms operate simultaneously: the presence of truly surviving core species (mostly *Firmicutes*) and the visibility of low-abundance DNA signals arising from cells broken down by digestion. The Shannon index reflects the balance between species distribution and species richness. The average Shannon values for the CN group are higher than those for GO. This indicates that the starter yogurt microbiota has a balanced species distribution and represents the stable ecosystem expected in fermented products. Shannon values for the GO group are significantly lower than those for CN. This decrease can be explained by the suppression of some probiotic species during the GO stage and by environmental factors (enzymes, pH changes) that destabilize species distribution. The G2 group shows a wide variation in the Shannon distribution. Some samples have high Shannon values, while others have very low ones. This indicates that although the number of species increases at the end of the digestion simulation, community equilibrium is re-established in very different ways, specific to each sample. The inverse Simpson index reflects a stronger dominance effect. The CN group has a higher and narrower distribution. This indicates that species dominance is lower in CN samples, suggesting a more balanced community. The GO group exhibits a low and tighter distribution. This shows that a few species become dominant in the GO stage, narrowing diversity. The G2 group has the widest range (approx. 0.5–7). The alpha diversity violin plot clearly highlights three key situations. The CN group presents a more balanced, stable fermented ecosystem with higher alpha diversity. The GO group shows that the gastrointestinal environment undergoes microbial contraction in the initial stage, with low diversity and a high dominant-species effect. The G2 group forms a heterogeneous, sample-dependent, variable ecosystem restructured at the phylum and genus level. Some products retain digestive resistance while others lose significant diversity.

**Figure 5:**
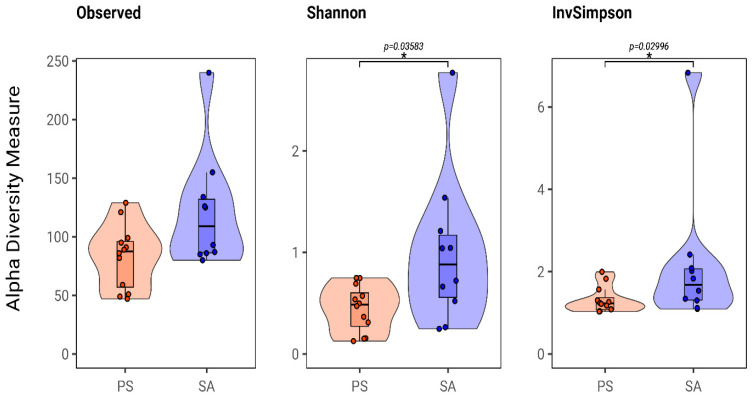
Alpha diversity violin plot at the genus level comparing PS and SA yogurt groups (* *p* < 0.05).

SA samples exhibit greater richness and uniformity than PS samples, reflecting the ecological impact of multi-variety probiotic sachet supplementation. PS samples cluster within a narrow range of diversity, consistent with fermentation using controlled starter cultures. Comparison of genus-level alpha diversity of PS and SA groups using Observed richness, Shannon, and Inverse Simpson indices is shown with violin plot graphs (Figure 5). The findings reveal that the two different production approaches significantly alter the microbial ecosystem structure. The Observed index shows that the SA group has a broader distribution and significantly higher species richness than the PS group. Species richness in the PS group products is clustered within a narrower range (~50–130), indicating that starter cultures form a standard, predictable, and microbially homogeneous structure. In the SA group, however, the dramatic increase in species richness in some samples (~50–240) suggests that probiotic sachets introduce broad microbial diversity to the product, combining with the endogenous flora in the traditional yogurt matrix to create a more heterogeneous ecosystem. Although this increased richness does not necessarily translate into greater biological activity, it effectively demonstrates that SA yogurts provide a broader probiotic repertoire. According to the Shannon index, the SA group differs significantly from the PS group (*p* = 0.04). This finding indicates that SA yogurts offer a more balanced microbial community, with probiotic species added via sachets being more evenly distributed not only numerically but also in relative abundance. Furthermore, the significant dominance of species in PS yogurts, with *Lactobacillus delbrueckii* and *Streptococcus thermophilus* showing high dominance in starter culture yogurts, lowers the Shannon index. This result shows that industrial starter cultures are strong in fermentation stability, aroma-texture standardization, and controlled microbial ecology, but are limited in microbial diversity. In the Inverse Simpson index, the SA group shows statistically significantly higher values than the PS group (*p* = 0.03). This indicates that no single species becomes excessively dominant in SA yogurts, the community is more balanced and competitive, and probiotic mixtures can coexist. In contrast, InvSimpson is lower in the PS group due to the dominance of dominant starter bacteria. This indicates that the community structure is based on a narrow probiotic core.

**Figure 6:**
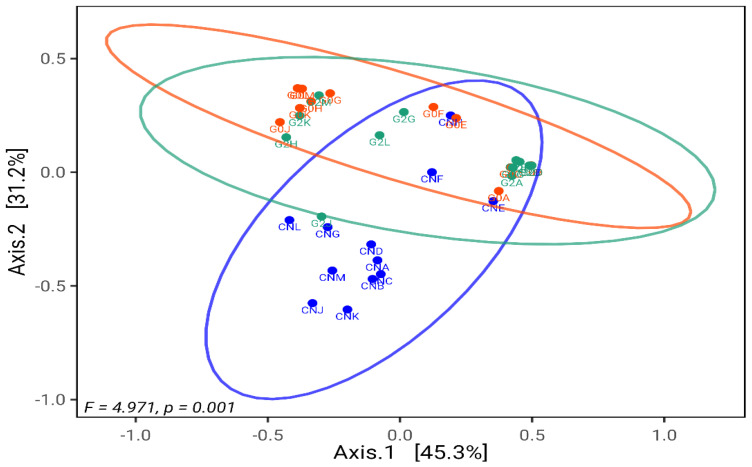
PCoA graph based on Bray–Curtis difference for CN, GO, and G2 samples.

Principal coordinate analysis reveals significant clustering among starter yogurt samples (CN), gastrointestinal entry samples (GO), and exit samples (G2). This demonstrates strong community restructuring throughout the digestive stages. PERMANOVA confirms significant group separation (*p* = 0.001). Bray–Curtis-based PCoA analysis (Figure 6) also shows significant divergence in microbial community structure among the CN, GO, and G2 groups. The first two axes explain 76.5% of the variance (Axis 1: 45.3%; Axis 2: 31.2%), surpassing what is typically observed in fermented-food microbiome studies. The PERMANOVA test (F = 4.971, *p* = 0.001) confirms a highly significant group difference. In the PCoA plot, CN samples (CNA–CNM) form a tight cluster. This indicates that the microbial composition of starter yogurts is highly reproducible, homogeneous, and stable. Fermentation appears controlled and dominated by specific species. From a food microbiology perspective, this shows that industrial yogurt production has achieved microbial standardization. GO samples diverge significantly from the CN cluster and spread over a wider area. This suggests a rebalancing of species due to chemical and enzymatic effects in the mouth phase, new interspecies competition, activation or suppression of probiotic species, and a shift in the GO cluster away from CN on both axes. Community composition changes at all taxonomic levels. The G2 group forms a third cluster, separate from the CN and GO clusters. This suggests that digestive simulation shifts the yogurt microbiota toward a new balance, influenced by factors such as pH drop, bile salts, proteolytic enzymes, and mixing. The G2 samples are more tightly clustered than GO but less than CN. This means that surviving probiotic species in G2 remain present, but their abundances vary across samples. A new “post-digestion core microbiota” has likely formed, reshaping along the *Lacticaseibacillus*–*Lactobacillus*–*Streptococcus* axis.

**Figure 7:**
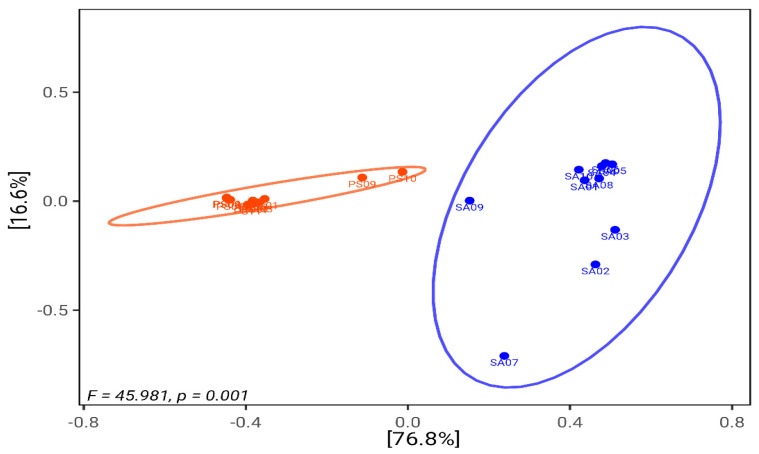
PCoA graph based on Bray–Curtis difference comparing PS and SA yogurt groups.

Figure 7 shows a sharp compositional divergence between tightly clustered starter culture yogurts (PS) and pouch-fortified yogurts (SA), which span a wider range. This demonstrates significant taxonomic heterogeneity introduced by probiotic pouches (PERMANOVA *p* = 0.001). The PCoA plot in Figure 7 shows a dramatic difference in microbial community structure between PS and SA at the genus level. The first two axes explain 93.4% of the total variance (Axis 1: 76.8%; Axis 2: 16.6%), a rate rarely seen this high in fermented product microbiome studies, indicating extremely strong ecological divergence between the groups. The PERMANOVA result (F = 45.981, *p* = 0.001) confirms that this divergence is statistically highly significant. PS samples (PS01–PS12) are tightly clustered in the graph and are located close to each other. This situation demonstrates that the microbiota of starter culture yogurts has a highly predictable, reproducible, low-variance, and technologically stable community structure. From a fermentation product science perspective, this structure is consistent with the strong dominance capacity of industrial starter cultures and the high standardization of fermentation conditions. SA samples (SA01–SA10) form a separate cluster from the PS group, but within themselves, they are spread over a wide area, exhibiting a more dispersed distribution and high ecological heterogeneity in microbial composition. This reflects differences in species content and viability among probiotic sachets. The species composition of each probiotic sachet varies. Therefore, the SA group has a wider microbial diversity from the outset. In addition, the combination of the endogenous microbiota of the traditional yogurt matrix with the sachets creates competition, adaptation, and coexistence among sachet-derived species and the traditional yogurt flora. This also causes the SA group to show a wider distribution compared to the PS group. The sharp separation of PS and SA groups along Axis 1 indicates that the community structure forms completely different ecosystems in terms of species richness, species abundance, dominant species, and taxonomic composition. This divergence is not merely a statistical difference but points to the existence of two biologically distinct probiotic ecological architectures.

## 4. Discussion

This study compared yogurts produced with starter cultures to traditional yogurts supplemented with probiotic sachets. Both product groups were also evaluated in an in vitro gastrointestinal system model to investigate the effects of the digestive process on microbial composition. The study’s overall findings show that both the production approach and gastrointestinal simulation conditions significantly alter the yogurt microbiota. When examining the alpha diversity and phylum-level composition data of yogurts produced with starter cultures, it was observed that they presented a highly homogeneous, low-variance, and predictable microbiota profile.

The Shannon diversity index accounts for the total number of taxa (richness) and the proportion (abundance) of each taxon in the sample. The higher the community diversity and the more evenly distributed the species, the higher the Shannon index. In contrast, the Chao1 index estimates the total number of species in each sample and provides a good representation of species with low abundance [16]. The observation of narrow distributions in the Observed, Shannon, and Inverse Simpson indices confirms that fermentation is tightly controlled by starter bacteria. It has been reported that Shannon and Simpson diversity indices differ significantly among fermented food groups, with milk-based fermented products such as yogurt and kefir exhibiting lower diversity than vegetable-based fermented products [17]. Maughan et al. [16] used Shannon and Chao1 indices to assess bacterial alpha diversity in retail kefir samples and found no statistically significant difference between groups in terms of the Shannon index. Similarly, although water kefirs showed lower average species richness in the Chao1 analysis, this difference was not statistically significant. The same study reported that some samples had high Shannon indices, while others showed high Chao1 values, indicating the presence of rare species.

In the PCoA analysis, the tight clustering of the PS group indicates a high level of genetic and taxonomic standardization of the microbial ecosystem. In contrast, yogurts with added probiotic sachets showed significantly higher, more heterogeneous values across all three indices. This situation demonstrates that the different probiotic species present in the sachets combine with the traditional yogurt matrix to create a multi-source and more resilient microbial ecosystem. The complete separation and wider spread of the SA group from the PS group in the PCoA analysis confirms that these ecosystems are genetically and functionally differentiated. The significant PERMANOVA result (*p* = 0.001) indicates that the divergence between the two production approaches is both statistically significant and biologically meaningful.

*Streptococcus thermophilus* strains isolated from traditional Turkish yogurts have been reported to exhibit high genetic diversity, and the 13 isolates examined belonged to different sequence types. Phylogenetic analyses revealed that *Streptococcus thermophilus* strains isolated from traditional Turkish yogurts are genetically distinct from strains of European origin [18].

Phylum-level distributions show that *Firmicutes* (*Bacillota*) dominance is maintained in both product types; this represents the characteristic fermenting bacteria of yogurt (*Lactobacillus* and *Streptococcus* spp.). However, the detection of *Actinomycetota* (especially *Bifidobacterium*), *Bacteroidota*, and low levels of *Pseudomonadota* in certain samples in the SA group indicates that probiotic sachets increase species intermingling. A comprehensive study of various fermented foods found that the *Firmicutes* phylum is dominant across all product groups and that lactic acid bacteria form the core of the microbial community [17]. The *Bacillota* dominance observed in our study’s yogurt samples is consistent with *Firmicutes*-dominant microbial profiles reported in 16S rRNA-based metagenomic analyses of commercial kefirs in Türkiye [19]. Furthermore, Gölbaşı et al. [20] reported that the *Firmicutes phylum* was dominant in over 97% of yogurt samples produced by traditional and back-slopping methods, while other phyla, such as *Bacteroidota* and *Proteobacteria*, were represented at low relative abundances. It has been reported that the *Lactobacillales* order and lactic acid bacteria (LAB) genera are abundant in milk-based fermented products such as yogurt and kefir and are among the main groups shaping the microbial structure of fermented foods [17].

The tight phylum distribution observed in starter yogurts provides advantages in terms of technological stability, sensory consistency, and shelf life, while the wide variation observed in sachet-added products offers a richer functional profile but may lead to greater variability in production standardization. In analyses performed at the phylum level, the *Bacillota* (*Firmicutes*) phylum was determined to be dominant in all samples. This finding is consistent with previous metagenomic studies reporting LAB dominance in fermented milk products. In particular, the dominance of the Firmicutes phylum has been frequently reported in 16S rRNA-based analyses performed on commercial kefir and yogurt products [19]. Similarly, the high proportion of *Bacillota* in the starter samples in this study confirms the decisive role of starter cultures in the fermentation process.

At the order level, the *Lactobacillales* order was dominant across all samples. This situation can be explained by the fact that *Lactobacillus* and *Streptococcus* species, commonly used in yogurt production, gain a competitive advantage by rapidly acidifying the environment during fermentation. Schillinger et al. [21] reported that the dominance of LAB species in probiotic yogurts arises from their acid tolerance and interactions with the milk matrix. In this context, the high *Lactobacillales* ratio observed in the input samples in our study is consistent with the literature. The *Lactobacillales* dominance observed in yogurt samples in this study is consistent with previous studies reporting that *Lactobacillus acidophilus* and *Lactobacillus johnsonii* groups are commonly used in probiotic yogurts [21]. It has been reported that the Lactobacillus genus is the most dominant bacterial genus in yogurt samples, followed by *Streptococcus* [20]. Yegin et al. [19] reported that *Lactococcus*, *Streptococcus*, and *Lactobacillus* species are the dominant LAB groups in industrial kefirs. Similarly, the high detection rate of *Lactobacillales* in yogurt samples in our study confirms LAB dominance originating from starter cultures. It has been reported that *Bifidobacterium* species are generally found in low abundance in commercial kefirs and are undetectable in some samples [19]. This explains the emergence of *Bifidobacteriales* as a secondary but functionally important group in our study. Palmnäs-Bédard et al. [17] reported that the addition of probiotic bacteria to some yogurts increased measured diversity and richness. They stated that after correction for multiple comparisons, no difference was found in the inverse Simpson estimates of alpha diversity.

In this study, the microbial community structure at the entry and exit stages of the gastrointestinal system in yogurt samples produced using starter cultures was evaluated using 16S rRNA amplicon sequencing. The results showed that the yogurt microbiota initially exhibited a relatively homogeneous LAB-dominated structure but underwent substantial compositional restructuring after gastrointestinal transit. Comparison of the G0 and G2 samples demonstrated that the digestive simulation exerted strong selective pressure on the yogurt microbiota. Although species richness and phylum diversity were relatively high in the G0 phase, diversity decreased markedly in G2 samples, resulting in an almost entirely Firmicutes-dominated microbial profile. This shift was mainly associated with the effects of low gastric pH, bile salts, proteolytic enzymes, mechanical mixing, and ionic stress, which favored the survival of acid- and bile-tolerant species. Under these conditions, species such as *Lacticaseibacillus rhamnosus*, *Lactobacillus delbrueckii*, and *Streptococcus thermophilus* remained dominant, whereas more sensitive taxa, particularly *Bifidobacterium* spp., were substantially reduced in G2 samples. Similar species-specific differences in gastrointestinal resistance have previously been reported in probiotic dairy products and in vitro digestion models [21,22].

The strain-dependent differences observed after gastrointestinal digestion may be associated with several intrinsic resistance mechanisms. Acid- and bile-tolerant LAB species such as *Lacticaseibacillus rhamnosus* and *Lactobacillus delbrueckii* are known to possess adaptive systems including F_0_F_1_-ATPase proton pumps, bile salt hydrolase activity, stress-response proteins, and membrane modifications that help maintain intracellular pH homeostasis under gastrointestinal stress conditions [23]. In addition, exopolysaccharide production and interactions with the milk matrix may provide physical protection against gastric acidity and bile exposure [21]. In contrast, Bifidobacterium species generally exhibit higher oxygen sensitivity and lower tolerance to acidic and bile-rich environments, which may explain their marked reduction after digestion. Competitive ecological interactions among strains may also contribute to microbial restructuring, as dominant LAB species can rapidly acidify the environment and suppress fewer resistant microorganisms. These mechanisms collectively suggest that post-digestion microbial composition is shaped not only by external gastrointestinal conditions but also by species-specific physiological and metabolic adaptation capacities [21,23].

Using the SHIME (Simulator of the Human Intestinal Microbial Ecosystem) model, Liu et al. [24] reported that *Bacteroides* species showed relatively stable distributions across biological replicates and represented a considerable proportion of the microbial population. The differences observed between their findings and the present study may be related to the use of human fecal microbiota in the SHIME model, whereas the current study focused on yogurt-associated microbial communities.

In their study comparing the selected probiotic properties of 9 strains of the *Lactobacillus acidophilus* group (6 *Lactobacillus acidophilus* and 3 *Lactobacillus johnsonii*) and 9 strains of the *Lactobacillus casei* group isolated from probiotic dairy products, Schillinger et al. [21] used an in vitro gastrointestinal system. They reported that *Lactobacillus acidophilus* strains were more tolerant of low pH (2.0) than *Lactobacillus* paracasei and *Lacticaseibacillus rhamnosus* strains, which rapidly lost viability after exposure to simulated gastric juice containing pepsin. The relative increase in *Bifidobacteriales* and bile-tolerant LAB species in the effluent samples can be attributed to the adaptive capacity of probiotic bacteria to gastrointestinal conditions. Schillinger et al. [21] reported that bile salt hydrolase activity, commonly detected especially in the *Lactobacillus acidophilus* group, may play a role in this adaptation. The species-specific survival differences observed in this study are consistent with previous findings showing that LAB strains do not exhibit equal resistance in in vitro gastrointestinal digestion models [23].

The formation of an independent G2 cluster, separate from the CN and GO groups in the Bray–Curtis-based PCoA analysis, indicates that the post-digestive microbial composition has reached a new ecological equilibrium. This structure clearly shows that the true probiotic effects of yogurt consumption should be understood through its G2 profile.

In their study characterizing the phenotype and genomes of LAB strains associated with human ileocecal mucosa using metagenomic sequencing and in vitro tests, Aziz et al. [23] found that *Enterococcus* and *Lactobacillus* strains showed tolerance of over 60% to human gastrointestinal system conditions such as lysozyme, gastric acidity, intestinal bile fluid, phenol, and osmotic pressure. In a simulated gastrointestinal digestion scenario, most strains-maintained viability rates above 80%.

Yegin et al. [19] reported that Shannon diversity indices in commercial kefirs varied widely, and evenness values differed between products. This supports the diversity–balance relationship observed in our study. Although observed richness data show an increase in species numbers in some G2 samples, this increase does not always indicate biological reproduction; detection of low-abundance DNA signals arising from cellular degradation during digestion may also contribute to this increase. Schillinger et al. [21] reported that *Lactobacillus delbrueckii* subsp. *bulgaricus* and *Streptococcus thermophilus*, known to have low resistance to gastric acid in vitro, may show high viability rates when consumed in a food matrix such as yoğurt.

Schillinger et al. [21] found that milk had a protective effect on strains less resistant to gastric acidity. They reported that bile salt hydrolase activity was detected in all strains of *Lactobacillus acidophilus*/*Lactobacillus johnsonii*, but not in strains of the *Lactobacillus casei* group. They concluded that all *Lactobacillus* could adhere to mucus-secreting HT29 MTX cells and human collagen type IV, fibrinogen, and fibronectin, but differences in the percentage of adhering bacteria were observed among different strains. This provides insight into the increase in G2-level diversity in our study, driven by differences in adhesion status and growth patterns among the microorganisms. Taken together, these findings are consistent with previous in vitro studies showing that lactic acid bacteria used in probiotic yogurts exhibit species-level selection and reorganization throughout the gastrointestinal system [21].

In the output samples obtained after gastrointestinal system transit, a relative decrease in the proportion of *Lactobacillales* was observed, while an increase was detected in the proportions of orders such as *Bifidobacteriales*, *Verrucomicrobiales*, and, in some samples, *Enterobacterales*. This change can be considered a result of the pressure exerted on the microbial community by the dynamic and selective environment of the gastrointestinal system. Studies using in vitro gastrointestinal system models have shown that the responses of LAB species to gastric and intestinal conditions can differ between species [22]. Similarly, genomic and phenotypic findings reported by Aziz et al. indicate that acid and bile tolerance of LAB strains is species-specific [23]. These species-specific resistance differences can be considered one possible mechanism explaining the microbial composition changes observed after gastrointestinal transit in our study.

Alpha diversity analyses revealed that the Shannon index varied widely among samples, and evenness values decreased, particularly in gastrointestinal output samples. The decrease in evenness values, despite the increase in species number, indicates that certain taxa have become dominant and that the community balance has shifted. This is consistent with the diversity–balance relationships reported in commercial kefir and fermented milk products [19]. Furthermore, the strong positive correlation found between the Shannon index and evenness reveals that increased diversity in microbial communities does not always guarantee ecological balance.

The relative increase in *Bifidobacterial* in output samples can be attributed to this group’s adaptation capacity to gastrointestinal conditions. Aziz et al. [23] reported that stress response genes (e.g., F_0_ F_1_-ATPase, Na^+^/H^+^ antiporters, and chaperone proteins) identified in *Bifidobacterium* and some *Lactobacillus* species play a role in acid and bile tolerance. These findings explain the persistence of secondary, yet functionally important, groups after gastrointestinal transit in our study.

Overall, this study reveals that the yogurt microbiota does not exhibit a stable structure throughout the gastrointestinal system, but rather reshapes itself at the community level from entry to exit. While previous studies have mostly focused on the viability and resistance of individual probiotic strains, this study, thanks to its community-level microbial profiling approach, offers an assessment encompassing the entire microbial community. In this respect, it provides new and complementary information at the community level to the literature on the behavior of fermented dairy products in the gastrointestinal system.

Overall, the findings of this study demonstrate that probiotic formulation strategy plays a decisive role in shaping yogurt microbiota composition and its resilience under gastrointestinal conditions. While starter culture yogurts exhibited a more homogeneous and technologically stable microbial structure, probiotic sachet supplementation increased microbial richness, ecological heterogeneity, and the survival potential of specific resistant LAB species during simulated digestion. The marked microbial restructuring observed between G0 and G2 samples confirms that gastrointestinal transit acts as a strong selective environment, favoring acid- and bile-tolerant taxa while reducing more sensitive microorganisms. These findings support the initial hypothesis that probiotic sachet supplementation enhances microbial diversity and alters post-digestive microbial survival dynamics. Importantly, this study highlights that the functional probiotic potential of yogurt should be evaluated not only based on the initial microbial composition of the product but also according to the microbial community that remains after gastrointestinal transit.

The strengths of the present study include an experimental design comparing microbial diversity in yogurt samples produced using probiotic starter cultures and those supplemented with probiotic sachets under a dynamic in vitro gastrointestinal system, high-resolution microbial profiling using ONT full-length 16S rRNA sequencing, and strong statistical group separation (PERMANOVA, *p* = 0.001). In addition, the comprehensive alpha- and beta-diversity analyses provided detailed insights into microbial community structure and compositional changes.

However, several limitations should be considered. Since microbial profiling was performed using 16S rRNA amplicon sequencing, which detects bacterial DNA regardless of cell viability, the method cannot directly distinguish between viable and non-viable microorganisms. Therefore, DNA originating from dead cells or extracellular sources may also contribute to the detected microbial composition, particularly in cases of increased diversity observed after digestion (e.g., G2 samples). Consequently, the findings should be interpreted as changes in microbial composition and persistence under simulated gastrointestinal conditions rather than direct evidence of bacterial survival.

In addition, several limitations associated with Oxford Nanopore Technology (ONT) should also be considered. Although ONT enables full-length 16S rRNA sequencing with improved taxonomic resolution, the platform may exhibit relatively higher sequencing error rates compared with short-read technologies. Potential amplification bias, variability in read quality, and challenges in distinguishing closely related taxa may influence taxonomic classification accuracy, particularly for low-abundance microorganisms. Furthermore, 16S rRNA-based sequencing provides taxonomic information but does not directly assess microbial function or metabolic activity.

Additional study limitations include the relatively limited sample size, the absence of initial viable counts of probiotic sachets and starter cultures, and the inability of the in vitro gastrointestinal system to fully reproduce the complexity of real gastrointestinal physiology. Furthermore, threats to the study include the lack of standardization of probiotic sachet contents available on the market, the potential for rapid microbiome alterations due to storage and processing variations, and the possibility of low-level contamination from environmental DNA sources.

Despite these limitations, this study is significant because it provides a basis for future functional investigations using shotgun metagenomic sequencing and metabolomics, supports in vivo validation of probiotic resistance, and contributes to the development of microbiome-based quality control strategies for probiotic yogurt products.

## 5. Conclusions

This study reveals that two different probiotic strategies used in yogurt production have multifaceted and profound effects on the in vitro gastrointestinal microbial community structure. When the results are evaluated holistically: starter culture yogurts are technologically more stable products with high homogeneity and predictable microbial structure.

Yogurts with added probiotic sachets offer higher species richness and diversity but have more variable microbial profiles. The in vitro gastrointestinal system largely eliminates starter product differences, creating a new core microbiota dominated only by resistant species. From the consumer’s perspective, the real probiotic benefit is determined more by the species that can survive post-digestion than by the initial composition of the product. These findings provide an important reference point for probiotic product development, yogurt technology, clinical nutrition, and microbiota research; in particular, they show that probiotic species selection, dosage, and formulation strategies should be designed considering post-digestion ecosystem behavior.

## Figures and Tables

**Table 1:** Genus-level alpha diversity indices for CN, GO, and G2 samples.

	Observed	Chao1	se.chao1	ACE	se.ACE	Shannon	InvSimpson
CNA	224	224	0	224	6.276	1.209	2.224
CNB	131	131	0	131	5.581	855	1.587
CNC	58	58	0	58	3.404	692	1.568
CND	117	117	0	NaN	NaN	1.502	2.582
CNE	57	57	0	57	3.603	756	1.693
CNF	132	132	0	NaN	NaN	2.342	5.44
CNG	85	85	0	85	4.406	0.16	1.039
CNH	53	53	0	53	3.639	723	1.882
CNJ	86	86	0	86	4.534	828	2.063
CNK	71	71	0	71	4.059	103	1.023
CNL	116	116	0	116	5.378	1.243	2.881
CNM	123	123	0	123	5.41	1.328	2.992
G0A	59	59	0	59	3.827	748	1.568
G0B	91	91	0	91	4.586	155	1.038
G0C	82	82	0	82	4.329	498	1.263
G0D	95	95	0	95	4.57	151	1.036
G0E	49	49	0	49	3.411	692	1.827
G0F	51	51	0	51	3.554	749	1.996
G0G	79	79	0	79	3.984	507	1.37
G0H	87	87	0	87	4.169	722	1.537
G0J	125	125	0	125	5.495	1.044	2.016
G0K	93	93	0	93	4.098	522	1.3
G0L	126	192.3	24.895	200.714	7.804	247	1.092
G0M	80	80	0	80	3.735	264	1.117
G2A	121	121	0	121	4.75	572	1.302
G2B	129	129	0	129	5.44	311	1.085
G2C	47	47	0	47	3.408	472	1.238
G2D	89	89	0	89	4.337	127	1.029
G2E	86	86	0	86	4.636	538	1.214
G2F	99	173.545	33.002	164.097	6.972	366	1.177
G2G	133	133	0	133	5.607	1.786	3.234
G2H	240	366.037	38.307	349.049	10.482	1.211	2.414
G2J	134	134	0	134	5.599	663	1.341
G2K	86	86	0	NaN	NaN	1.539	2.084
G2L	85	85	0	NaN	NaN	2.77	6.832
G2M	155	155	0	155	5.257	1.041	1.831
***p***-value	0.05 *	0.09	0.42	0.01 *	0.03 *	0.26	0.38

Observed richness, Chao1, ACE, Shannon, and Inverse Simpson indices are presented for starter yogurts (CN), gastrointestinal simulation inlet samples (GO), and outlet samples (G2). The data demonstrate substantial reductions and restructuring of microbial diversity following gastrointestinal exposure, with selective survival of acid- and bile-tolerant genera. PS: Probiotic starter culture, SA: Sachet added. * *p* < 0.05.

**Table 2:** Genus-level alpha diversity indices for PS (probiotic starter) and SA (sachet added) yogurt groups.

	Observed	Chao1	se.chao1	ACE	se.ACE	Shannon	InvSimpson
PS01	59	59	0	59	3.827	748	1.568
PS02	91	91	0	91	4.586	155	1.038
PS03	82	82	0	82	4.329	498	1.263
PS04	95	95	0	95	4.57	151	1.036
PS05	121	121	0	121	4.75	572	1.302
PS06	129	129	0	129	5.44	311	1.085
PS07	47	47	0	47	3.408	472	1.238
PS08	89	89	0	89	4.337	127	1.029
PS09	49	49	0	49	3.411	692	1.827
PS10	51	51	0	51	3.554	749	1.996
PS11	86	86	0	86	4.636	538	1.214
PS12	99	173.545	33.002	164.097	6.972	366	1.177
SA01	87	87	0	87	4.169	722	1.537
SA02	240	366.037	38.307	349.049	10.482	1.211	2.414
SA03	125	125	0	125	5.495	1.044	2.016
SA04	93	93	0	93	4.098	522	1.3
SA05	126	192.3	24.895	200.714	7.804	247	1.092
SA06	80	80	0	80	3.735	264	1.117
SA07	134	134	0	134	5.599	663	1.341
SA08	86	86	0	NA	NA	1.539	2.084
SA09	85	85	0	NA	NA	2.77	6.832
SA10	155	155	0	155	5.257	1.041	1.831
*p*-value	0.09	0.11	0.47	0.05 *	0.16	0.04 *	0.03 *

The table compares richness and community balance metrics between two production strategies. SA samples display higher richness and more even genus distribution than PS samples, indicating that probiotic sachets introduce broader microbial communities into the yogurt matrix. PS: Probiotic starter culture, SA: Sachet added. * *p* < 0.05.

## Data Availability

The original contributions presented in the study are included in the article, further inquiries can be directed to the corresponding author.
